# Comparison of faculty and student perceptions of sexual and gender minority content in a preclerkship medical curriculum

**DOI:** 10.1186/s12909-023-04925-7

**Published:** 2023-12-19

**Authors:** Benjamin Crosby, Isabelle M. Gell-Levey, Janet Monroe, Carl G. Streed, Jennifer Siegel, Erin E. Carter, Nat Mulkey, Ann C. Zumwalt

**Affiliations:** 1 Boston University Chobanian & Avedisian School of Medicine, Boston, MA USA; 2 Departments of Pediatrics & Child Neurology, Barrow Neurological Institute at Phoenix Children’s Hospital, Phoenix, AZ USA; 3 Department of Medicine, Beth Israel Deaconess Medical Center, Boston, MA USA; 4 Center for Transgender Medicine and Surgery, Boston Medical Center, Boston, MA USA; 5 Transgender Health Program, Massachusetts General Hospital, Boston, MA USA; 6 Division of Urology, University of Utah School of Medicine, Salt Lake City, Utah USA; 7 Department of Psychiatry, The University of Vermont Medical Center, Burlington, VT USA; 8 Department of Anatomy & Neurobiology, Boston University Chobanian & Avedisian School of Medicine, Boston, MA USA

**Keywords:** Undergraduate medical education, Sexual and gender minorities, Curriculum assessment, Sexual orientation, Gender identity, LGBTQ +

## Abstract

**Background:**

Sexual and gender minority (SGM) persons experience stark health disparities. Efforts to mitigate disparities through medical education have met some success. However, evaluations have largely focused on subjective perspectives rather than objective measures. This study aimed to quantify Boston University School of Medicine’s sexual and gender minority (SGM) education through surveys of course directors (CDs) and medical students regarding where SGM topics were taught in the preclerkship medical curriculum. Responses were compared to identify concordance between faculty intention and student perceptions regarding SGM education.

**Methods:**

A cross-sectional survey was distributed to preclerkship CDs and current medical students in Spring 2019 and 2021, respectively, regarding where in the mandatory preclerkship curriculum CDs deliberately taught and where first- and second-year students recalled having learned 10 SGM topic domains.

**Results:**

64.3% of CDs (*n* = 18), 47.0% of the first-year class (*n* = 71), and 67.3% of the second-year class (*n* = 101) responded to the surveys. Results indicate that, as anticipated, deliberate SGM teaching correlates with greater student recall as students recalled topics that were reported by CDs as intentionally taught at a significantly higher rate compared to those not intentionally taught (32.0% vs. 15.3%; *p* < 0.01). Students most commonly recalled learning SGM-related language and terminology, which is likely partly but not entirely attributed to curricular modifications and faculty development made between distribution of the faculty and student surveys, indicating the importance of all faculty being trained in appropriate SGM terminology and concepts. Discordance between faculty intention and student recall of when topics were taught reveals opportunities to enhance the intentionality and impact of SGM teaching.

**Conclusions:**

Students perceive and recall SGM content that is not listed as learning objectives, and all faculty who utilize this material in their teachings should receive foundational training and be thoughtful about how information is framed. Faculty who intentionally teach SGM topics should be explicit and direct about the conclusions they intend students to draw from their curricular content.

**Supplementary Information:**

The online version contains supplementary material available at 10.1186/s12909-023-04925-7.

## Background

Sexual and gender minority (SGM) patient populations, including lesbian, gay, bisexual, transgender, and queer (LGBTQ +) persons, experience stark health disparities [1]. Despite these known disparities, the SGM health content taught in most medical curricula is limited, with undergraduate medical education (UME) schools in the United States and Canada reporting a median of only 5 h dedicated to LGBT-related curricular content across the entire curriculum [2]. Efforts to rectify this educational gap across undergraduate and graduate medical education curricula have been inconsistent, resulting in “a lack of required standardized training competencies… and limited faculty experience" (p. 275) [2]. Consequently, the majority of medical students report feeling unprepared to address the health needs of SGM patients, likely contributing to the ongoing healthcare disparities experienced by this population [3]. There is widespread desire among both medical faculty and trainees to learn more about SGM health [4].

Targeted efforts to address the lack of improved SGM training in undergraduate medical education have met some success. A focused 4-h session on transgender health implemented among preclerkship students resulted in 73% of student respondents reportedly feeling more prepared to provide care for transgender patients [5]. 80% of fourth-year medical students who participated in a series of standardized patient cases that explored aspects of transgender patient healthcare reported feeling more prepared to provide care to SGM patients afterwards [6]. Incorporation of SGM-inclusive content in an endocrinology course showed a 67% reduction in student discomfort towards providing transgender care, while 85% of students who gained clinical exposure to transgender health through a new medicine elective reported “high” knowledge regarding transgender patient management [7, 8]. These studies illustrate that intentional teaching of SGM topics can have substantial impact on learners’ feelings of preparedness to care for this population.

While targeted lessons can be useful, at the institutional level the most relevant outcome is learner preparedness at the conclusion of the curriculum when learners graduate to embark in their practice of medicine. Appreciating that longitudinal education is more powerful than single lessons, a number of institutions have intentionally infused SGM content across entirety of the curriculum, either for a subset of interested students [9] or for the entire student body [10, 11]. Institutions working to improve trainee preparation to care of this population must examine and continually work to improve the training experienced by these learners across the entirety of the medical curriculum.

Our institution has the goal of ensuring our learners are well prepared to care for SGM patients. To understand the quality of our SGM curriculum and how it can be improved, we first examined the existing state of where SGM topics are taught in our curriculum. This effort involved examining both the formal curriculum as designed by the educators as well as the experience of the curriculum from the student perspective. Both perspectives were examined because curricular design is a complex process that requires alignment between faculty intention, faculty development, instructional methods, student assessment, and student recall [12]. There can be a difference between what is intended to be taught by curricular leadership, what is delivered in the classroom by individual lecturers, and what is actually received by learners [13]. As students do not necessarily recall all information they have been taught [14], thorough assessment of the existing curriculum requires evaluating not only what faculty report teaching, but also what students perceive they have learned. The majority of prior literature has compared student and faculty impressions on effective teaching methods [15, 16], though there remains a dearth of literature that specifically compares faculty and student perception of the same curricular content. At present, we are only aware of one other study that compared student and faculty perceptions of UME content, which was conducted in the United Kingdom and did not specifically assess traditionally excluded medical education content such as SGM health [17]. As such, we sought to characterize how recently incorporated SGM topics were being perceived by medical students in comparison to faculty intention, specifically: (1) Do students recall SGM content intentionally incorporated into the curriculum? (2) What SGM topics are not recalled by the students, if any? (3) Is there content that the students recall learning in courses where it was not intentionally taught, and if so, what is it?

In this study, we examine how student recall of SGM topics in the preclerkship curriculum correlates with course director (CD) reports of where these topics were intentionally built into that curriculum. Based on preliminary observations, we hypothesize that students will recall some but not all SGM content that was intentionally taught by faculty. We also hypothesize that concordance and discordance between faculty and student perspectives will provide insight into the effectiveness of intended curricula and what makes SGM content memorable for students. Understanding where these perceptions do and do not align will better inform and direct educators on methods to maximize the impact of SGM healthcare content in undergraduate medical education.

## Methods

This study was approved by the Boston University Medical Campus Institutional Review Board (H-40948).

### Data collection

The SGM-Curriculum Assessment Tool (SGM-CAT, Supplement 1) was developed in 2018 through the BUSM Gender and Sexual Diversity Vertical Integration Group (GSD VIG)and has been described in a prior publication [18]. In brief, the SGM-CAT assesses coverage of 12 core SGM topics distilled from the Association of American Medical Colleges’ (AAMC) recommended SGM competency domains for a UME curriculum (Table 1) [19]. The SGM-CAT was distributed between May 2019 to June 2019 to all CDs of mandatory preclerkship courses and clerkships to assess where and how SGM content is taught across the medical curriculum. When clarification about individual responses was needed, GSD VIG members contacted individual CDs by email or in person.

**Table 1:** SGM topics assessed in the SGM-CAT medical student survey. Items marked with an asterisk (*) were asked as two separate questions on the faculty survey. Otherwise, the faculty survey was the same

Language and terminology related to sexual and gender diversity
Taking an inclusive sexual history for LGBTQ + individuals including about their partners and practices
Cancer screening for sexual or gender minority patients
Sexually transmitted infection screening or prevention of HIV (PrEP) in sexual and gender minority patients^*^
Contraception or family/fertility planning for sexual and gender minorities
Mental health needs in sexual and gender minority patients
Development of sexual and gender identities over the lifespan (including psychology, anatomy, genetics, etc.)
Gender-affirming care (hormone therapy, surgical, etc.)
Healthcare disparities/inequities or health policy issues related to sexual and gender minority populations^*^
Mistrust of healthcare professionals by individuals who identify as sexual and/or gender minorities

The SGM-CAT was subsequently streamlined by combining two topics together to assess 10 SGM topics (Table 1). This modified survey (Supplement 2) was distributed to current BUSM students between March 2021 to April 2021. At this time the first year students had completed all but one of the M1 modules and second year students had completed all M1 and M2 courses. First year students assessed all M1 courses except Endocrine/Reproduction and M2 students assessed all M1 and M2 courses. The survey was distributed using Qualtrics software (QualtricsⓇ XM, Provo, UT, USA). The survey was advertised through a weekly newsletter and reminders on BUSM-affiliated social media. After survey completion, respondents were given the option to voluntarily provide an email address in order to be entered into a raffle for one of 25 Visa gift cards valued at $35.

The structures of the faculty and student surveys were similar in that the participants were asked to identify where specific SGM topics were taught. For the faculty survey, the CD identified the course or clerkship they were responsible for and then identified which of the 12 SGM topic domains (Table 1) they teach in their course or clerkship. They were also given the opportunity to elaborate on why topics are or are not taught in their courses (not reported in this study) (Supplement 1). On the student survey, students first identified their stage in the curriculum at the time of the survey. Based on that information, the survey logic offered them all courses they should have completed at that stage of the curriculum. Courses were listed by year, and students were asked about the same topics listed in Table 1: “Which of the following topics relating to sexual and gender minority populations were addressed in your (year) courses?” (Supplement 2).

### Analysis

This study includes analysis of the faculty survey and the M1 and M2 student survey response data pertaining to the mandatory preclerkship courses. All second-year student (MS2) responses were included, as MS2s had completed all preclerkship courses at the time of the survey. First-year student (MS1) data was analyzed for first year courses with the exception of the first-year Endocrinology/Reproduction course, which these students had not completed at the time of survey completion.

After preliminary analysis of the survey results, courses were excluded from further study if there appeared to be no coverage of any SGM topics based on both CD and student responses. This was defined as being when the CD reported no coverage of any SGM topics in their course *and* students similarly did not recall any SGM topics in that course, using a threshold of less than 20% student respondents recalling relevant content in that course. Based on these criteria, of the 18 preclerkship courses surveyed there was no coverage of any SGM topic coverage in the following 9 courses: M1 Cellular Foundations of Medicine, M1 Immunology, M1 Neurology, M1 Cardiovascular, M1 Respiratory, M1 Gastrointestinal/Nutrition, M1 Renal, M2 Introduction to Careers in Medicine, M2 Renal. These courses are excluded from further reporting in this study.

Students reported whether or not they recalled learning a specific SGM topic (Table 1) in a particular course. For all included courses, these recall rates were calculated by adding the number of students who recalled learning the topic in that course and dividing it by the number of survey respondents who completed that course at the time of the survey. These recall rates were then compared to whether the CD reported that the topic was taught in that course using ANOVA and independent student t-tests (two tailed, alpha = 0.05). Average recall rates between MS1 and MS2 respondents across all courses were analyzed using independent student t-tests (two-tailed, alpha = 0.05). Results were analyzed using SPSS 26 (IBM Corp. Released 2019. IBM SPSS Statistics for Mac, Version 26.0. Armonk, NY: IBM Corp).

During the years covered in this study, there were ongoing curricular and faculty development efforts at our institution in an effort to increase inclusivity of SGM health topics. Because of this, course syllabi from 2018–19 and 2020–21 were comprehensively reviewed for modifications related to the SGM survey topics to evaluate whether modifications were made to course content between distribution of the faculty and subsequent student surveys.

## Results

The majority of preclerkship CDs completed the faculty survey (18 of 28 courses; 64.3%) [18]. Approximately half of the student body completed the student survey (329 of 729 students; 45.1%), with respondents from all four curricular years. Responses analyzed in this study include 71 MS1 students (47.0% of M1 class) and 101 MS2 students (67.3% of M2 class), or 52.3% of the total survey responses. There was no significant difference in overall recall rates between MS1 and MS2 cohorts (*p* = 0.85).

In the courses in which SGM topics appeared, topics that were reported by faculty as being intentionally taught were recalled by an average of 32.0% of students as having learned the topic in that part of the curriculum. For all SGM topics that faculty reported not intentionally teaching, an average of 15.3% of students recalled learning the topic in that part of the curriculum (Table 2). Collectively, students recalled SGM topics reported by CDs as explicitly taught significantly more often than those not intentionally taught (*p* < 0.01).

**Table 2:**
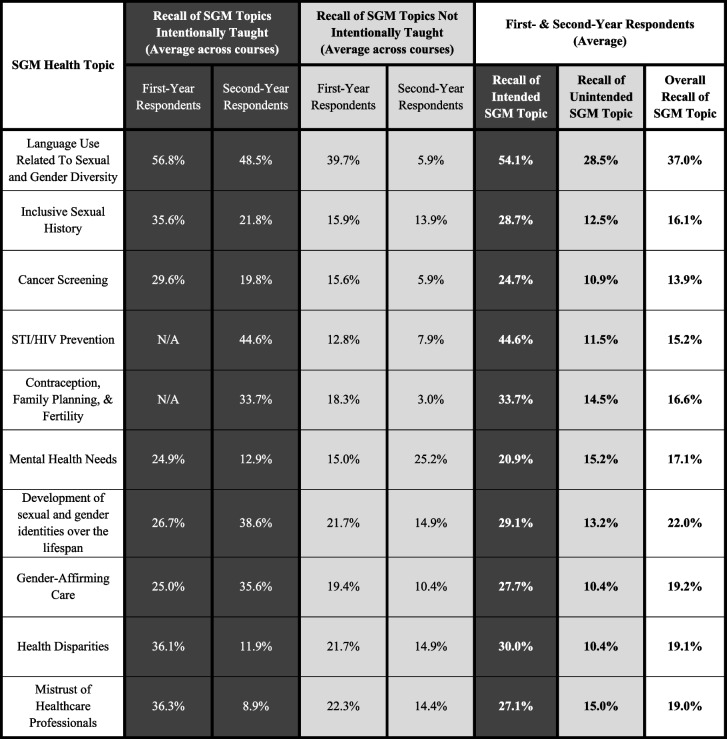
Percentages of first- and second-year student overall recall by SGM topic being taught in the listed course. Responses were stratified by topics faculty intended to teach (black boxes), did not intend to teach (gray boxes), and the average percentage of all topics. White boxes indicate the average percentages of students who indicated recalling an SGM topic being taught in all courses, regardless of faculty indication

In some cases, CDs reported not teaching a topic, yet students recalled learning the topic. For example, students recalled learning SGM Language and Terminology at rates of 49.0%, 45.5%, and 41.8% in Body Structures (gross anatomy), Endocrinology/Reproduction, and Essentials of Public Health, respectively, despite CDs not indicating having intentionally taught that topic. Other topics that students recalled learning about despite faculty not intending to teach include SGM Contraception, Family Planning & Fertility in Genomic Medicine (33.1%) and Endocrinology/Reproduction (39.6%), SGM Mental Health Needs in Psychiatry (48.5%), and Mistrust of Health Professionals in Essentials of Public Health (36.6%) (Table 3). Notably, the post hoc comparison of 2018–19 and 2020–21 materials did reveal differences in how some material was presented in the M1 Body Structures, M1 Genomics, and M1 Endocrine/Reproduction courses in an effort to be more inclusive of SGM topics. Examples include the adoption of gender-inclusive phrases (“pregnant people”, “gestational carrier/parent”, “breastfeeding parents”) and explicit disclosures regarding how “male/female” are used to denote aspects of biological sex and genetics rather than gender.

**Table 3:**
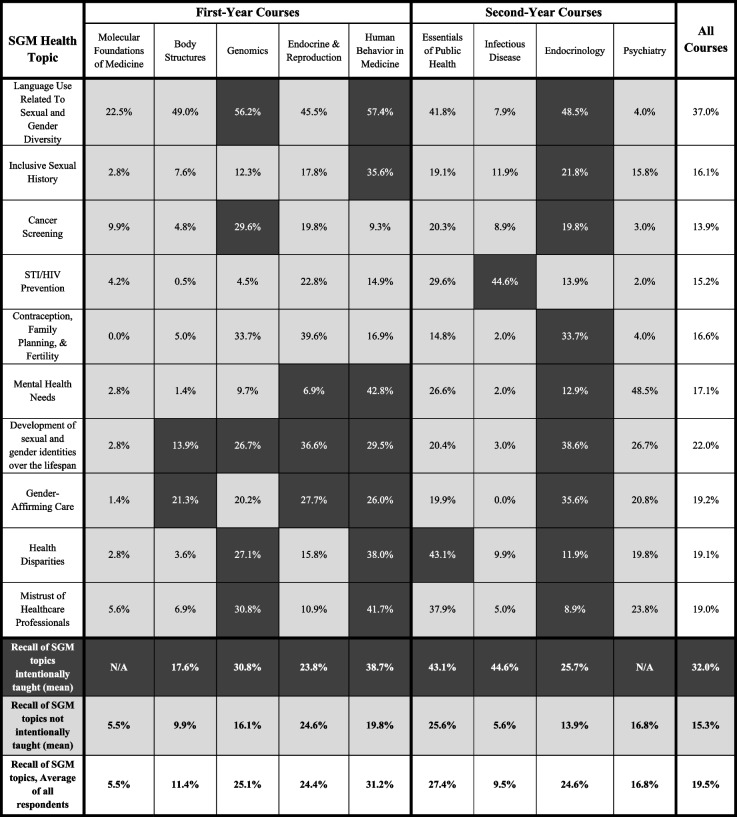
Percentages of students who recall individual SGM topics being covered in each analyzed preclerkship course. Responses are stratified by topics faculty intended to teach (black boxes), did not intend to teach (gray boxes), and the average percentage of all topics (white boxes). MS1 and MS2 responses are averaged

Conversely, some topics that CDs reported teaching were recalled by a relatively low percentage of students (< 20.0%). Both the first-year Endocrinology/Reproduction and second-year Endocrinology CDs reported teaching SGM Mental Health Needs; however, only 6.9% and 12.9% of students, respectively, recalled this topic being taught in those courses. The second-year Endocrinology CD reported intentionally teaching 9 of the 10 select SGM topics, excluding only STI/HIV prevention, yet relatively few students reported having learned about Cancer Screening (19.8%), Health Disparities (11.9%), or Mistrust in Health Professionals (8.9%) in this course. Additionally, while Development of Sexual and Gender Identity Across the Lifespan was indicated as being intentionally taught in Body Structures, only 13.9% of students recalled learning it (Table 2).

## Discussion

Our study aimed to compare preclerkship faculty intention to teach sexual and gender minority (SGM) topics with student recall of having learned these topics. The results of this study provide insight into how educators can maximize the impact of SGM content in an undergraduate medical curriculum.

### Alignment of faculty intention and student recall

As anticipated, in this study significantly more students recalled learning SGM topics in courses where faculty intended to teach the topic, compared to SGM topic areas that faculty did not intend to teach. Students appeared to particularly recall material that was explicitly included in learning objectives and lecture material, as demonstrated by the relatively high rate of recall of Inclusive Sexual History Taking in the Human Behavior in Medicine course which incorporated a focused lecture on the topic. Current research in teaching and learning indicates that the most effective way of learning is through testing, in contrast to passive study [20, 21]. This background in combination with our results suggests that medical educators who wish to improve SGM teaching should include dedicated time for SGM topics and identify testable learning objectives in order to increase student retention of SGM content.

### Misalignment of faculty intention and student recall

Whileit is not particularly surprising that students recall information that was intentionally taught, more revealing are the insights that come from examining when student recall does not reflect what faculty intended to teach in their curricula. In our study, students both recalled learning information that faculty did not intend to teach and did not recall learning information that faculty did teach.

#### Students recalled information that was not intentionally taught

There were a number of topics that students recalled learning in courses where the CDs did not intend to teach them. The adjustments made in three M1 courses to include more SGM-inclusive language and framing between the administration of the two surveys likely explains why students recalled use of SGM Language and Terminology in these courses in contrast to faculty responses from 2019. There are still several other student-faculty response discrepancies that were not elucidated upon course content review, and there are two potential explanations for how students could recall information that was not actually taught. First, they may have assumed the topic was included in certain courses given the nature of the course itself. For example, students recalled learning about SGM Mental Health Needs in the second-year Psychiatry course and recalled SGM Contraception and Family Planning in the first-year Endocrine/Reproduction course, despite CDs not reporting having taught these topics and confirmed upon review of the course content in 2021. Students likely recalled the topics because it was a reasonable assumption that they would have learned about them in these courses. This discrepancy highlights current gaps and provides an opportunity for course leadership to bolster SGM teaching where it should logically exist.

A second explanation for student recall of information that was not intentionally taught could be a difference in perspectives between what students and faculty mean when they consider a topic “addressed.” From a faculty perspective, *addressing a topic* implies the topic was explicitly built into the course with associated content, learning objectives, and potentially assessment. Students, on the other hand, may have interpreted the same survey wording as asking if an SGM topic came up at all during the course, such as during discussions, clinical case vignettes, or spontaneously during lectures. Students observe the use of the language, and are less likely to parse in hindsight whether or not topics discussed in class were listed as learning objectives. For example, there were relatively high numbers of students who reported learning SGM Language and Terminology in three courses—Body Structures, Endocrinology/Reproduction, and Essentials of Public Health—in which the CDs reported no intention to teach it (Table 3). Follow-up investigation with these CDs revealed that they did use SGM language (e.g. cisgender, transgender, intersex) to teach about other topics, but their intention was not to explicitly teach students about SGM terminology. These insights have major implications regarding the importance of faculty development and training to support *all*faculty in their knowledge and comfort with speaking on biological sex, gender, and SGM terminology, regardless of individuals’ perception of whether or not the topic is relevant to their content [22]. Given that SGM topics can come up in teaching despite it not being a primary intention of the course, it is imperative to ensure that this “unintentional curriculum” is as thoughtful, sensitive, and accurate as possible. Future studies comparing faculty and student perceptions of any curricula should be proactive and intentional about defining what is meant by any content being “addressed” or not.

#### Students did not recall information that was intentionally taught

There were also a number of areas in which students did not recall topics that the faculty intended to convey (Table 2). These disconnects reveal opportunities for faculty to more deliberately emphasize SGM topics they intend to teach. For example, the Body Structures (gross anatomy) director (author AZ) reported that SGM Development Across the Lifespan was taught in that course; however, relatively few students recalled learning this topic in that course. Follow-up investigation determined that the CD defined urogenital embryology, including differences in sexual development, as falling under this topic. However, neither the topic’s relevance to SGM health nor its connection to intersex anatomy was explicitly emphasized in the course. Therefore, learners did not recognize the connection of urogenital embryology to SGM Development Across the Lifespan, particularly for intersex individuals. Revealing this discrepancy to the CD created an opportunity to make changes in future iterations of the course to more strongly emphasize the connection. These insights emphasize that faculty who intend to teach SGM topics should be explicit and direct about the conclusions they intend students to draw from their curricular content to ensure that the intended SGM learning objectives are achieved.

### Overall recall

**Fig. 1:**
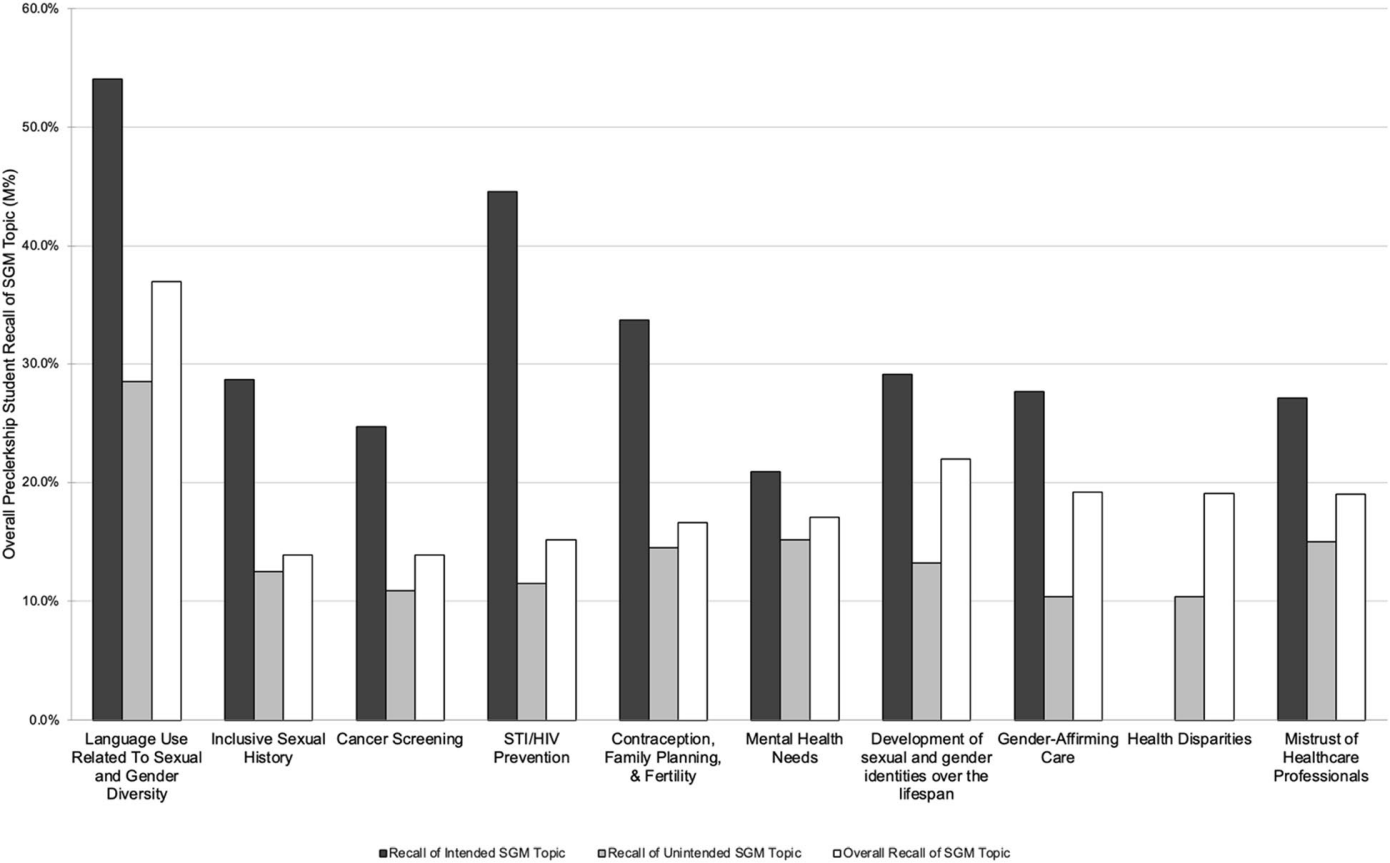
Mean percentage of students who recall SGM topics that were intentionally vs not intentionally taught by faculty, all preclerkship courses combined. Responses are stratified by topics faculty intended to teach (black boxes), did not intend to teach (gray boxes), and the average percentage of all topics (white boxes)

Overall, the student recall of when they learned SGM topics was low. With a few notable exceptions, even topics intentionally included in the curriculum by faculty were recalled by less than 30% of the students as being taught in that course (Fig. 1). It is unlikely that student recall of the content itself is low, as the student survey form did not question recall of specific facts about SGM health and most studies of longer-term retention of basic science facts indicate 66–75% of learners recall facts after 1–2 years [16, 23]. Rather, it is more likely that students do not recall when in the curriculum they learned the topics. Notably, overall recall rates of the MS1 and MS2 cohorts were statistically equivalent, thus implying that while the recall rates were low, the rates are an accurate reflection of long-term retention of this information.

Nevertheless, the results of this study do suggest an opportunity to investigate what topics students recall at the end of a preclerkship curriculum. Many papers describe a lack of forward momentum in medical education reform [15, 24, 25], and there also appears to be a delay between bridging recognition of the importance of a curricular issue and resolving it with curricular change. For example, in a medical student perspective article on ethics teachings, students noted a discrepancy between faculty appreciation of medical ethics and actual teaching efforts behind the topic. Learning objectives were often ambiguous and treated as afterthoughts to lectures, and content was delivered in a non-interactive manner despite the plasticity of the subject [26]. A parallel can be readily drawn to the topic of SGM health, which has received an increased amount of attention in recent years and is continuing to evolve as it is actively incorporated into medical curricula. The relatively low recall by students of when they learned certain topics may imply that faculty could do a more effective job at conveying the material they intend to teach. These efforts are important and meaningful, as demonstrated by studies of SGM-inclusive medical curricula which consistently report positive impacts on learners’ attitudes and self-perceived competence [13, 14, 27–29].

### Limitations

There are certain important limitations to acknowledge in this study. The survey was cross-sectional rather than longitudinal, relying on self-reported student recall about previous courses rather than asking about each course as the curriculum progressed. Since the survey asked students to recall topic coverage some months after they had originally taken each course, students may have forgotten or recalled an altered impression of when they learned certain topics. Additionally, the student survey was administered two years after the CD survey, so some of the discrepancies may be explained by changes to course content during that time. As noted above, between the CD survey and the student survey some courses began intentionally defining the difference between sex and gender and describing how each type of term would be used in the course. While this practice was not defined on the CD survey as being an “SGM topic,” it is possible that students recalled it as such.

Given the retrospective nature of this study, there is also the possibility that the recall of delivered content was inaccurate. For example, the faculty survey was answered by CDs, but these CDs were not necessarily the individuals responsible for the relevant lectures. There is a possibility that the content taught did not align with what CDs reported as having been taught to students. Course directors at our institution are expected to closely monitor the content delivered in their courses, so while possible, it is unlikely that content was delivered of which they were not aware. Similarly, the student survey was completed by approximately half of the student body with individually varying backgrounds and prior knowledge in SGM-related topics. It is likely that medical students with a stronger foundation in or affinity to SGM health were both more likely to answer the survey and had a better recall of curricular teachings, which may have skewed these results. We are unable to substantiate this potential impact on survey results as medical student attitudes towards SGM topics were not assessed in this study.

Additionally, the findings in this paper do not comprehensively reflect the entire BUSM preclerkship curriculum because 35.7% of preclerkship CDs did not respond to original faculty survey [18] and because courses that showed no faculty intention or student recall of SGM topics were omitted from analysis and follow-up. Finally, this survey was administered at only one medical school, which may limit the generalizability of the results to other medical schools.

## Conclusions

BUSM students and faculty were generally in agreement about where SGM topics were covered in the preclerkship curriculum. The discrepancies between CD intention and student recall provide interesting insights into opportunities to improve how SGM curricula are taught. Faculty sometimes unintentionally address certain SGM topics, in particular language and terminology, whereas other SGM topics that faculty intend to teach are going unnoticed by students. These discrepancies emphasize that faculty must be explicit and clear about the relevance of basic science topics to SGM health, and that all medical faculty should be thoroughly trained and comfortable with SGM language and terminology, as students observe language use regardless of faculty intention to highlight the topic. When faculty do intend to teach SGM topics, they should be explicit about the message they intend learners to take away from the material, rather than relying on learners to draw their own conclusions.

It appears that conscious, deliberate efforts to use SGM language and terminology correctly and to include SGM topics in the curriculum are noticed by students, and such efforts likely improve the overall impact of the curriculum on learner’s preparedness to treat this population. Future research should examine student recall of SGM content over time, and the impact of faculty efforts on this recall. Longer term studies should examine the most impactful design of SGM curricula to train the next generation of physicians to provide proper care to their sexual and gender minority patients.

### Supplementary Information

**Additional file 1.**

**Additional file 2.**

## Data Availability

The datasets used and/or analyzed during the current study are available from the corresponding author on reasonable request.

## Abbreviations

SGM: Sexual and gender minority
CDs: Course directors
LGBTQ +: Lesbian, gay, bisexual, transgender, queer, and others
UME: Undergraduate medical education
BUSM: Boston University Chobanian & Avedisian School of Medicine
SGM-CAT: Sexual and Gender Minority-Curriculum Assessment Tool
GSD VIG: Gender and Sexual Diversity Vertical Integration Group
AAMC: Association of American Medical Colleges
M#: Year of curriculum (e.g. M1: first year course)
MS#: Medical student, class year (e.g. MS1: medical student, first-year class)
