# Supplementary material for: Comparison of faculty and student perceptions of sexual and gender minority content in a preclerkship medical curriculum
Source: BMC Med Educ. 2023 Dec 19;23:973. doi: 10.1186/s12909-023-04925-7 (PMC10731801; doi:10.1186/s12909-023-04925-7)
Supplement: Supplementary file 1 — Additional file 1. [file 12909_2023_4925_MOESM1_ESM.docx]

**Supplement #1**. SGM-CAT survey by Zumwalt et al. (<https://doi.org/10.1097/ACM.0000000000004203>), distributed among medical school faculty to assess where SGM topics delineated by the AAMC SGM Competency Objectives were taught in the curriculum.

**SGM Topics in BUSM Curriculum - Faculty**

**Start of Block: Default Question Block**

In this survey you will be asked a series of questions about curriculum related to sexual and gender minority populations. Please answer to the best of your knowledge about one course or clerkship at a time. If you run more than one course/clerkship, please fill out this survey separately for each one.

**Definitions
*Sexual minority*:** Individual whose sexual identity, orientation or practices differ from the majority of the surrounding society. Primarily used to refer to lesbian, gay, and bisexual individuals but also includes asexual and pansexual.  ***Gender minority*:** Individual whose gender identity (person's sense of their own gender) differs from the binary designations of cis-male and cis-female. Includes transgender, nonbinary, and intersex individuals.

**Your name**

**________________________________________________________________**

**Name of course or clerkship**

**________________________________________________________________**

**What year students participate in this course or clerkship?**

**▢ MS1**

**▢ MS2**

**▢ MS3**

**▢ MS4**

**Approximately how many students participate in this experience each year?
If this is a mandatory clerkship, write 100%**

**________________________________________________________________**

**Do you address topics related to gender identity (person's sense of their own gender) in your course / clerkship in any way?**

**o Yes**

**o No**

***Do you address topics related to gender identity (person's sense of their own gender) in your course? = Yes***

**Please elaborate how gender identity is addressed in your curriculum.**

**________________________________________________________________**

**________________________________________________________________**

**________________________________________________________________**

**________________________________________________________________**

**________________________________________________________________**

***Do you address topics related to gender identity (person's sense of their own gender) in your course? = No***

**What are the reasons or barriers to doing so?**

**________________________________________________________________**

**________________________________________________________________**

**________________________________________________________________**

**________________________________________________________________**

**________________________________________________________________**

**Do you address topics related to sexual identity in your course / clerkship in any way?**

**o Yes**

**o No**

***Do you address topics related to sexual identity in your course / clerkship in any way? = Yes***

**Please elaborate how sexual identity is addressed in your curriculum.**

**________________________________________________________________**

**________________________________________________________________**

**________________________________________________________________**

**________________________________________________________________**

**________________________________________________________________**

***Do you address topics related to sexual identity in your course / clerkship in any way? = No***

**What are the reasons or barriers to doing so?**

**________________________________________________________________**

**________________________________________________________________**

**________________________________________________________________**

**________________________________________________________________**

**________________________________________________________________**

**Please indicate which of the following topics are addressed in your course or clerkship.**

|  | **All students/ mandatory** | **Some students / depends on location** | **No** | **Not applicable** |
| --- | --- | --- | --- | --- |
| **Appropriate language use related to sexual and gender diversity** | **o** | **o** | **o** | **o** |
| **Components of an inclusive sexual history for LGBTQ+ individuals (partners, practices)** | **o** | **o** | **o** | **o** |
| **Cancer screening for sexual or gender minority patients** | **o** | **o** | **o** | **o** |
| **Sexually transmitted infection screening in sexual and gender minority patients** | **o** | **o** | **o** | **o** |
| **Prevention of HIV (e.g. Truvada)** | **o** | **o** | **o** | **o** |
| **Contraception or family/fertility planning for sexual and gender minorities** | **o** | **o** | **o** | **o** |
| **Mental health needs in sexual and gender minority populations** | **o** | **o** | **o** | **o** |
| **Development of sexual and gender identities over the lifespan (including psychology, anatomy, genetics, etc.)** | **o** | **o** | **o** | **o** |
| **Gender affirming care (hormone therapy, surgical, etc.)** | **o** | **o** | **o** | **o** |
| **Health or healthcare disparities / inequities related to sexual and gender minority populations** | **o** | **o** | **o** | **o** |
| **Mistrust of healthcare professionals by individuals who identify as sexual and/or gender minorities** | **o** | **o** | **o** | **o** |
| **Health policy, legal or ethical issues related to the care of gender or sexual minorities** | **o** | **o** | **o** | **o** |

**Please elaborate on your answers above as desired.**

**________________________________________________________________**

**________________________________________________________________**

**________________________________________________________________**

**________________________________________________________________**

**________________________________________________________________**

**Please share anything about your curriculum related to gender and sexual minority populations that you have not yet had the opportunity to share elsewhere in this survey.**

**________________________________________________________________**

**________________________________________________________________**

**________________________________________________________________**

**________________________________________________________________**

**________________________________________________________________**
