# Supplementary material for: Comparison of faculty and student perceptions of sexual and gender minority content in a preclerkship medical curriculum
Source: BMC Med Educ. 2023 Dec 19;23:973. doi: 10.1186/s12909-023-04925-7 (PMC10731801; doi:10.1186/s12909-023-04925-7)
Supplement: Supplementary file 2 — Additional file 2. [file 12909_2023_4925_MOESM2_ESM.docx]

**Supplement #2.** Cross-sectional survey streamlined from the 2019 SGM-CAT faculty survey by Zumwalt et al. (<https://doi.org/10.1097/ACM.0000000000004203>), distributed among a medical student body to assess where in the curriculum respondents recalled having learned 10 SGM topic domains.

**BUSM LGBTQI+ Curriculum Survey**

**Start of Block: Introduction and Consent**

The purpose of this study is to characterize the quantity and quality of medical education about the healthcare of Sexual and Gender Minority (SGM) populations in the BUSM curriculum. This study specifically focuses on the student perspective of the inclusion of these topics in their medical training. Please read the following consent form that discusses the basic information and overview of the study, benefits and risks of participating in this study, confidentiality, and provides research team contact information. The survey is expected to take 20 minutes. By participating in this survey, you have the opportunity to be selected to receive one of 25 Visa Gift Cards with a value of $35. Upon completion of the survey, you will be redirected to a secondary page to enter your name and Boston University email address to enter the drawing. These responses will not be linked to data gathered in the main student survey in any way. Winners will be drawn within one week of the completion of the data collection period. If selected, you will be contacted via email to access the Visa Gift Card. By agreeing to be in this research, you are indicating that you have read the consent form and that you voluntarily agree to participate in this research study.

**Definitions**

***Sexual and gender minority*:** term to encompass populations that are included in the acronym “LGBTQI+” (lesbian, gay, bisexual, transgender, queer/questioning, and intersex) as well as individuals whose gender identity and or sexual orientation varies.

***HIV prophylaxis*:** a preventative regimen that includes two anti-viral medications and are regularly taken by people who have not contracted HIV. Brand name examples include Truvada and Descovy.

**What is the year of medical school for which you last took courses?**

- **M1**
- **M2**
- **M3**
- **M4**

**Doc/ICM Which clinical skills courses did you take?**

|  |  |
| --- | --- |
| **M1** | **▼ Doctoring 1 or ICM-1** |
| **M2** | **▼ Doctoring 1 or ICM-1** |

**Which of the following M3 clerkships have you taken? Please include your current course.**

- **Surgery**
- **Obstetrics & Gynecology**
- **Pediatrics**
- **Family Medicine**
- **Internal Medicine**
- **Psychiatry**
- **Neurology**
- **Emergency Medicine**
- **Radiology**

**What selective did you take during M3? Do not select courses that you enrolled in as a M4.**

- **Radiology**
- **Emergency Medicine**
- **Enrichment**

**Have you taken, or are you currently enrolled in Geriatrics?**

- **Yes**
- **No**

**How comfortable are you in providing care to sexual and gender minority patients?**

- **Comfortable**
- **Somewhat comfortable**
- **Neutral**
- **Somewhat uncomfortable**
- **Uncomfortable**

**Has your level of comfort with providing care to sexual and gender minority patients changed as a result of your education at BUSM?**

- **My comfort has increased**
- **My level of comfort has not changed**
- **My comfort has decreased**
- **I do not know**

**Which of the following topics relating to sexual and gender minority populations were addressed in your M1 courses? (1 of 2)**

|  | **terminology related to sexual and gender diversity** | **taking an inclusive sexual history** | **cancer screening** | **STI screening or HIV prophylaxis** | **contraception, fertility, or family planning** | **none of these topics were addressed** |
| --- | --- | --- | --- | --- | --- | --- |
| **MFoM** |  |  |  |  |  |  |
| **CFoM** |  |  |  |  |  |  |
| **Body Structures** |  |  |  |  |  |  |
| **Neurosciences** |  |  |  |  |  |  |
| **Genomic Medicine** |  |  |  |  |  |  |
| **Immunology** |  |  |  |  |  |  |
| **Cardiovascular** |  |  |  |  |  |  |
| **Respiratory** |  |  |  |  |  |  |
| **Renal** |  |  |  |  |  |  |
| **GI/Nutrition** |  |  |  |  |  |  |
| **Endocrinology & Reproduction** |  |  |  |  |  |  |
| **HBM** |  |  |  |  |  |  |
| **EPH** |  |  |  |  |  |  |
| **Doctoring 1** |  |  |  |  |  |  |
| **ICM 1** |  |  |  |  |  |  |
| **IP 1** |  |  |  |  |  |  |

**Which of the following topics relating to sexual and gender minority populations were addressed in your M1 courses? (2 of 2)**

|  | **mental health needs** | **development of sexual and gender identities over the lifespan** | **gender-affirming care** | **health policy or healthcare disparities** | **mistrust of healthcare professionals** | **none of these topics were addressed** |
| --- | --- | --- | --- | --- | --- | --- |
| **MFoM** |  |  |  |  |  |  |
| **CFoM** |  |  |  |  |  |  |
| **Body Structures** |  |  |  |  |  |  |
| **Neurosciences** |  |  |  |  |  |  |
| **Genomic Medicine** |  |  |  |  |  |  |
| **Immunology** |  |  |  |  |  |  |
| **Cardiovascular** |  |  |  |  |  |  |
| **Respiratory** |  |  |  |  |  |  |
| **Renal** |  |  |  |  |  |  |
| **GI/Nutrition** |  |  |  |  |  |  |
| **Endocrinology & Reproduction** |  |  |  |  |  |  |
| **HBM** |  |  |  |  |  |  |
| **EPH** |  |  |  |  |  |  |
| **Doctoring 1** |  |  |  |  |  |  |
| **ICM 1** |  |  |  |  |  |  |
| **IP 1** |  |  |  |  |  |  |

**Which of the following topics relating to sexual and gender minority populations were addressed in your M2 courses? (1 of 2)**

|  | **terminology related to sexual and gender diversity** | **taking an inclusive sexual history** | **cancer screening** | **STI screening or HIV prophylaxis** | **contraception, fertility, or family planning** | **none of these topics were addressed** |
| --- | --- | --- | --- | --- | --- | --- |
| **Foundations** |  |  |  |  |  |  |
| **Infectious Diseases** |  |  |  |  |  |  |
| **Pulmonary** |  |  |  |  |  |  |
| **Cardiovascular** |  |  |  |  |  |  |
| **Rheumatology** |  |  |  |  |  |  |
| **GI** |  |  |  |  |  |  |
| **Renal** |  |  |  |  |  |  |
| **Endocrinology** |  |  |  |  |  |  |
| **Reproduction** |  |  |  |  |  |  |
| **Neurology** |  |  |  |  |  |  |
| **Psychiatry** |  |  |  |  |  |  |
| **Dermatology** |  |  |  |  |  |  |
| **Oncology** |  |  |  |  |  |  |
| **Hematology** |  |  |  |  |  |  |
| **Doctoring 2** |  |  |  |  |  |  |
| **ICM 2** |  |  |  |  |  |  |
| **IP 2** |  |  |  |  |  |  |

**G-M2-2 Which of the following topics relating to sexual and gender minority populations were addressed in your M2 courses? (2 of 2)**

|  | **mental health needs** | **development of sexual and gender identities over the lifespan** | **gender-affirming care** | **health policy or healthcare disparities** | **mistrust of healthcare professionals** | **none of these topics were addressed** |
| --- | --- | --- | --- | --- | --- | --- |
| **Foundations** |  |  |  |  |  |  |
| **Infectious Diseases** |  |  |  |  |  |  |
| **Pulmonary** |  |  |  |  |  |  |
| **Cardiovascular** |  |  |  |  |  |  |
| **Rheumatology** |  |  |  |  |  |  |
| **GI** |  |  |  |  |  |  |
| **Renal** |  |  |  |  |  |  |
| **Endocrinology** |  |  |  |  |  |  |
| **Reproduction** |  |  |  |  |  |  |
| **Neurology** |  |  |  |  |  |  |
| **Psychiatry** |  |  |  |  |  |  |
| **Dermatology** |  |  |  |  |  |  |
| **Oncology** |  |  |  |  |  |  |
| **Hematology** |  |  |  |  |  |  |
| **Doctoring 2** |  |  |  |  |  |  |
| **ICM 2** |  |  |  |  |  |  |
| **IP 2** |  |  |  |  |  |  |

**Which of the following topics relating to sexual and gender minority populations were addressed in your M3/4 clerkships? (1 of 2)**

|  | **terminology related to sexual and gender diversity** | **taking an inclusive sexual history** | **cancer screening** | **STI screening or HIV prophylaxis** | **contraception, fertility, or family planning** | **none of these topics were addressed** |
| --- | --- | --- | --- | --- | --- | --- |
| **Internal Medicine** |  |  |  |  |  |  |
| **Surgery** |  |  |  |  |  |  |
| **Family Medicine** |  |  |  |  |  |  |
| **Obstetrics & Gynecology** |  |  |  |  |  |  |
| **Psychiatry** |  |  |  |  |  |  |
| **Pediatrics** |  |  |  |  |  |  |
| **Neurology** |  |  |  |  |  |  |
| **Radiology** |  |  |  |  |  |  |
| **Emergency Medicine** |  |  |  |  |  |  |
| **Geriatrics** |  |  |  |  |  |  |

**Which of the following topics relating to sexual and gender minority populations were addressed in your M3/4 clerkships? (2 of 2)**

|  | **mental health needs** | **development of sexual and gender identities over the lifespan** | **gender-affirming care** | **health policy or healthcare disparities** | **mistrust of healthcare professionals** | **none of these topics were addressed** |
| --- | --- | --- | --- | --- | --- | --- |
| **Internal Medicine** |  |  |  |  |  |  |
| **Surgery** |  |  |  |  |  |  |
| **Family Medicine** |  |  |  |  |  |  |
| **Obstetrics & Gynecology** |  |  |  |  |  |  |
| **Psychiatry** |  |  |  |  |  |  |
| **Pediatrics** |  |  |  |  |  |  |
| **Neurology** |  |  |  |  |  |  |
| **Radiology** |  |  |  |  |  |  |
| **Emergency Medicine** |  |  |  |  |  |  |
| **Geriatrics** |  |  |  |  |  |  |

**Start of Block: Topic 1-Language and terminology**

**In your opinion, how well was language and terminology related to sexual and gender diversity addressed?**

***This question and the free responses below is repeated for each topic from the above grid that participants select***

|  | **Very Poor** | **Poor** | **Neutral** | **Good** | **Very Good** |
| --- | --- | --- | --- | --- | --- |
| **MFoM** |  |  |  |  |  |
| **CFoM** |  |  |  |  |  |
| **Body Structures** |  |  |  |  |  |
| **Neurosciences** |  |  |  |  |  |
| **Genomic Medicine** |  |  |  |  |  |
| **Immunology** |  |  |  |  |  |
| **Cardiovascular** |  |  |  |  |  |
| **Respiratory** |  |  |  |  |  |
| **Renal** |  |  |  |  |  |
| **GI/Nutrition** |  |  |  |  |  |
| **Endo/Repro** |  |  |  |  |  |
| **HBM** |  |  |  |  |  |
| **EPH** |  |  |  |  |  |
| **Doctoring 1** |  |  |  |  |  |
| **ICM 1** |  |  |  |  |  |
| **IP 1** |  |  |  |  |  |
| **Foundations** |  |  |  |  |  |
| **Infectious Diseases** |  |  |  |  |  |
| **Pulmonary** |  |  |  |  |  |
| **Cardiovascular** |  |  |  |  |  |
| **Rheumatology** |  |  |  |  |  |
| **GI** |  |  |  |  |  |
| **Renal** |  |  |  |  |  |
| **Endo** |  |  |  |  |  |
| **Repro** |  |  |  |  |  |
| **Neurology** |  |  |  |  |  |
| **Psychiatry** |  |  |  |  |  |
| **Dermatology** |  |  |  |  |  |
| **Oncology** |  |  |  |  |  |
| **Hematology** |  |  |  |  |  |
| **Doctoring 2** |  |  |  |  |  |
| **ICM 2** |  |  |  |  |  |
| **IP 2** |  |  |  |  |  |
| **Internal Medicine** |  |  |  |  |  |
| **Surgery** |  |  |  |  |  |
| **Family Medicine** |  |  |  |  |  |
| **Obstetrics & Gynecology** |  |  |  |  |  |
| **Psychiatry** |  |  |  |  |  |
| **Pediatrics** |  |  |  |  |  |
| **Neurology** |  |  |  |  |  |
| **Radiology** |  |  |  |  |  |
| **Emergency Medicine** |  |  |  |  |  |
| **Geriatrics** |  |  |  |  |  |

**Please elaborate on how language and terminology related to sexual and gender diversity was discussed.**

**________________________________________________________________**

**________________________________________________________________**

**________________________________________________________________**

**________________________________________________________________**

**________________________________________________________________**

**Did you notice any opportunities to address language and terminology related to sexual and gender diversity when they were not? If so, please elaborate.**

**________________________________________________________________**

**________________________________________________________________**

**________________________________________________________________**

**________________________________________________________________**

**________________________________________________________________**

**End of Block: Topic 1-Language and terminology**

**Start of Block: Topic 2- inclusive sexual history**
